# Direct Interaction of the N-Terminal Domain of Ribosomal Protein S1 with Protein S2 in *Escherichia coli*

**DOI:** 10.1371/journal.pone.0032702

**Published:** 2012-03-07

**Authors:** Konstantin Byrgazov, Salim Manoharadas, Anna C. Kaberdina, Oliver Vesper, Isabella Moll

**Affiliations:** Max F. Perutz Laboratories, Department of Microbiology, Immunobiology and Genetics, Center for Molecular Biology, University of Vienna, Vienna, Austria; New England BioLabs, United States of America

## Abstract

Despite of the high resolution structure available for the E. coli ribosome, hitherto the structure and localization of the essential ribosomal protein S1 on the 30 S subunit still remains to be elucidated. It was previously reported that protein S1 binds to the ribosome via protein-protein interaction at the two N-terminal domains. Moreover, protein S2 was shown to be required for binding of protein S1 to the ribosome. Here, we present evidence that the N-terminal domain of S1 (amino acids 1–106; S1_106_) is necessary and sufficient for the interaction with protein S2 as well as for ribosome binding. We show that over production of protein S1_106_ affects E. coli growth by displacing native protein S1 from its binding pocket on the ribosome. In addition, our data reveal that the coiled-coil domain of protein S2 (S2α_2_) is sufficient to allow protein S1 to bind to the ribosome. Taken together, these data uncover the crucial elements required for the S1/S2 interaction, which is pivotal for translation initiation on canonical mRNAs in Gram-negative bacteria. The results are discussed in terms of a model wherein the S1/S2 interaction surface could represent a possible target to modulate the selectivity of the translational machinery and thereby alter the translational program under distinct conditions.

## Introduction

A pivotal step in regulation of gene expression is the initiation of translation, more precisely, the initial interaction between the ribosome and the mRNA [1]. In *Escherichia coli* and most Gram-negative bacteria protein S1 is a key player that mediates the primary binding of the 30 S ribosomal subunit to the ribosome binding site (rbs) of mRNAs [2]. S1 represents the largest ribosomal protein with a molecular weight of 61.1 kDa. In particular, it is implicated in translation initiation complex formation on mRNAs comprising highly structured 5′-untranslated regions (UTR) [3], [4]. The protein interacts with a pyrimidine-rich region upstream of the Shine and Dalgarno (SD)-sequence [5] and was suggested to unwind RNA secondary structures [6], [7], thereby facilitating the positioning of the 30 S subunit in close proximity to the translational start site [8]. In contrast, S1 is dispensable for translation of leaderless mRNAs (lmRNAs) that start directly with the AUG codon thus lacking a 5′-UTR [9], [10].

**Figure 1:**
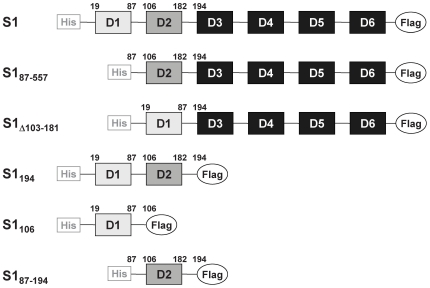
Schematic depiction of protein S1 and its variants used in this study. All protein variants were C-terminally FLAG-tagged to facilitate detection. For pull-down assays shown in Figure 4 and for protein purification, protein variants contained an additional N-terminal HIS-tag.

S1 is composed of six contiguous OB (oligonucleotide–oligosaccharide-binding) folds, the ‘so-called’ S1 domains, which are approximately 70 amino acids in size (Figure 1) [11]. Although structurally related these domains exhibit distinct functions (Figure 1): the two N-terminal domains (D1 and D2) are suggested to be involved in ribosome binding and interaction with the Qβ replicase [12]–[14]. Moreover, domain D2 was reported to play an essential role in the recognition and binding to tmRNA [15]. Domains D3–D6 were suggested to form an elongated RNA-binding domain that protrudes into the solvent [16]. Domains D3–D5 bind single stranded RNA [5], [13], [17], [18], whereas the most distal domain (D6; Figure 1) is involved in autogenous regulation of *rpsA* expression [19]. Recently, the functional specialization of the different domains has been supported by phylogenetic trees built from the alignment of domain sequences of S1 proteins derived from Gram-negative as well as Gram-positive bacteria [18].

Despite its essentiality in the process of translation initiation, to date there is no structure of the native protein S1, and moreover the protein is missing in the high resolution structures available for the *E. coli* ribosome. However, reconstitution experiments revealed that some proteins within the group of S2, S3, S9, S10, and S14 are important for assembly of S1 to the 30 S subunit [20]. Cross-linking studies suggested a potential localisation of S1 at the platform, between the main body and the head of the 30 S subunit, in close proximity to proteins S2, S10, and S18 [21]. More recently, this platform localisation was supported by the comparison of cryo-electron data of the 30 S ribosomal subunit of *E. coli* with an X-ray crystallographic structure of a 30 S subunit of *T. thermophilus* lacking S1 [22], which substantially underlined the notion of a direct interaction between proteins S1 and S2. Moreover, the observation that *E. coli* ribosomes lacking protein S2 are likewise devoid of protein S1 [10], [23] indicated that protein S2 is essential for binding of S1 to the 30 S ribosomal subunit. In addition, the formation of a stoichiometric complex of proteins S1 and S2 was reported [24], which is implicated in the regulation of the expression of the *rpsB*-*tsf* operon encoding ribosomal protein S2 and translation elongation factor EF-Ts [25].

The present study was conducted to gain insights into the binding mode of protein S1 to the ribosome. With the objective to determine structural features required for binding of the protein to the ribosome, we tested for assembly of different truncated protein S1 variants. Our results indicate that solely the N-terminal domain D1 (here referred to as protein S1_106_) is responsible and required for the interaction of S1 with the ribosome. Our data indicate that overexpression of the S1_106_ protein, representing the N-terminal S1 domain, inhibits translation of bulk mRNA whereas lmRNAs translation continues. Moreover, we verify that the direct interaction between domain D1 and ribosomal protein S2 is pivotal for binding of protein S1 to the ribosome.

## Results

### The N-terminal domain D1 of protein S1 is required for binding to the ribosome in vivo

**Figure 2:**
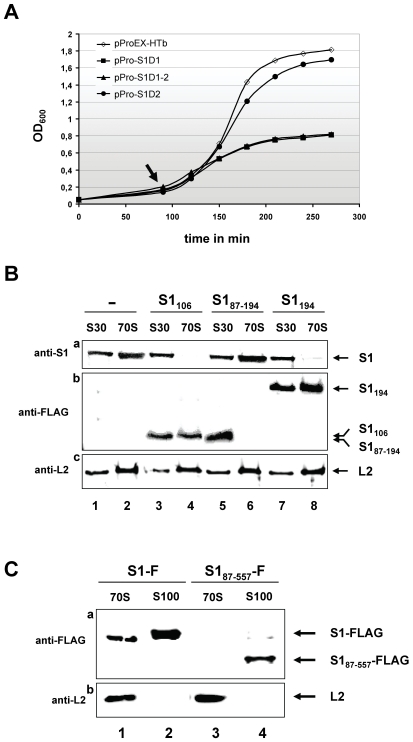
Protein S1_106_ affects *E. coli* growth by displacing native S1 from the ribosome. (**A**) In contrast to synthesis of protein S1_87–194_ (representing domain D2, -•-), synthesis of S1_106_ and S1_194_ (representing domains D1 (-▪-) and D1-2 (-▴-)) inhibits bacterial growth. *E. coli* strain JE28 harbouring plasmids pProEX-HTb (-◊-), pPro-S1D1F (-▪-), pPro-S1D2F (-•-) and pPro-S1D1-2F (-▴-) were grown in LB medium containing ampicilin (100 µg/ml) and kanamycin (20 µg/ml). At OD_600_ of 0.2–0.25 (indicated by an arrow) 50 µM IPTG was added to the cultures. Aliquots were withdrawn from each culture for ribosome preparation 1 hour upon induction. (**B**) Proteins present in S30 extracts (lanes 1, 3, 5, and 7) and 70 S ribosomes (lanes 2, 4, 6, and 8) prepared from cells without overexpression (lanes 1 and 2), and cells overexpressing S1_106_ (lanes 3 and 4), S1_87–194_ (lanes 5 and 6), or S1_194_ (Lanes 7 and 8) were separated on a 12.5% SDS-PAGE and presence of protein S1 and its variants on 70 S ribosomes was checked by western blot analysis using anti-S1 antibodies (panel a), anti-FLAG (panel b) and anti-L2 antibodies (panel c), which served as loading control. The positions of the respective proteins are indicated to the right. (**C**) The N-terminal domain of S1 is required for assembly to the ribosome. Equimolar amounts of HIS-tagged ribosomes (lanes 1 and 3) and ribosome free S100 extract (lanes 2 and 4) purified from *E. coli* strain JE28 overexpressing FLAG-tagged proteins S1 (lanes 1 and 2) and S1_87–557_, lacking domain D1 (lanes 3 and 4) were separated on a 12.5% SDS-PAGE. The presence of S1 and S1_87–557_ was determined by western blot analysis employing anti-FLAG antibodies (panel a) and anti-L2 antibodies (panel b), which served as loading control.

Previous studies indicated that the N-terminal fragment of protein S1 comprising domains D1 and D2 (protein S1_194_, Figure 1) is pivotal for ribosome binding [12], [13], [26]. However, based on the information of a phylogenetic tree built on alignments of protein S1 sequences from Gram-negative bacteria, domains D1 and D2 are suggested to have different roles in ribosome binding [18]. Therefore, the first aim of this study was to narrow down the interaction site between S1 and the ribosome. To distinguish, whether domain(s) D1 and/or D2 are required for ribosome binding, FLAG-tagged S1 variants comprising either domain D1 (S1_106_), domain D2 (S1_87–194_), or both domains D1–D2 (S1_194_) were overexpressed *in vivo*. *E. coli* strains JE28 [27] harbouring plasmids pPro-S1D1F, pPro-S1D2F, or pPro-S1D1-2F (Table 1) coding for the respective S1 fragments under control of the *trc* promoter were grown in LB broth at 37°C. At OD_600_ of 0.2 synthesis of S1 variants was induced by addition of 50 µM IPTG (Isopropyl-β-D-thiogalactopyranosid). As expected, synthesis of protein S1_194_ severely affected growth (Figure 2A) due to inhibition of protein synthesis, since binding of native S1 is prevented by the ribosome bound S1_194_ variant [12], [13]. This effect was mirrored by synthesis of protein S1_106_ (comprising solely domain D1) as cell growth was inhibited in a comparable manner. In contrast, synthesis of S1_87–194_ (representing domain D2) did not affect growth, already indicating that domain D2 is not involved in ribosome binding (Figure 2A). Concomitantly, the cells were harvested 60 minutes (min) upon induction and ribosomes were isolated to determine the assembly of the different S1 variants. As *E. coli* strain JE28 harbours a modified *rplL* gene encoding a HIS-tagged protein L7/L12 [27], 70 S ribosomes were purified employing Ni-NTA agarose as specified in Materials and Methods. Western blot analysis of ribosomal proteins employing anti-FLAG antibodies revealed the presence of proteins S1_194_ (Figure 2B, panel b, lane 8) and S1_106_ (Figure 2B, panel b, lane 4) on the ribosome *in vivo*. As expected, this binding severely reduced the amount of native protein S1 present on the ribosome (Figure 2B, panel a, lanes 4 and 8). In contrast, protein S1_87–194_ comprising domain D2 was not detected in the 70 S fraction (Figure 2B, panel b, lane 6), and consequently no reduction in amount of protein S1 on the ribosome was observed (Figure 2B, panel a, lane 6).

**Table 1:** Bacterial strains and plasmids used in this study.

	Genotype/Relevant features	Source/Reference
***E. coli*** ** strains:**		
JE28	MG1655::*rplL-his*	[27]
Tuner	F^−^ *ompT hsdS*_B_ (r_B_^−^ m_B_^−^) *gal dcm lacY1*	Novagen
Tuner(DE3)	F^−^ *ompT hsdS*_B_ (r_B_^−^ m_B_^−^) *gal dcm lacY1(DE3)*	Novagen
**Plasmids:**		
pKTplaccI	*cI-lacZ* fusion gene under lac promoter control	[28]
pProEX-HTb		Invitrogen
pProEX-S1F	pProEX encoding HIS- and FLAG-tagged protein S1	This study
pProEX-S1ΔD1F	pProEX encoding HIS- and FLAG-tagged protein S1_87–557_	This study
pProEX-S1ΔD2F	pProEX encoding HIS- and FLAG-tagged protein S1_Δ103–181_	This study
pProEX-S1D1-2F	pProEX encoding HIS- and FLAG-tagged protein S1_194_	This study
pProEX-S1D1F	pProEX encoding HIS- and FLAG-tagged protein S1_106_	This study
pProEX-S1D2F	pProEX encoding HIS- and FLAG-tagged protein S1_87–194_	This study
pPro-S1F	pProEX-S1F encoding FLAG-tagged protein S1	This study
pPro-S1ΔD1F	pProEX-S1ΔD1F encoding FLAG-tagged protein S1_87–557_	This study
pPro-S1ΔD2F	pProEX-S1ΔD2F encoding FLAG-tagged protein S1_Δ103–181_	This study
pPro-S1D1-2F	pProEX-S1D1-2F encoding FLAG-tagged protein S1_194_	This study
pPro-S1D1F	pProEX-S1D1F encoding FLAG-tagged protein S1_106_	This study
pPro-S1D2F	pProEX-S1D2F encoding FLAG-tagged protein S1_87–194_	This study
pPro-S1ΔD2F	pProEX-S1ΔD2F encoding FLAG-tagged protein S1_87–557_	This study
pET22b		Novagen
pET-ccS2	pET derivative encoding HIS-tagged protein S2α_2_	This study

### Protein S1 lacking the N-terminal domain D1 does not bind to the ribosome in vivo

To verify that only domain D1 is involved in interaction with the ribosome, the affinity of a truncated variant of S1 lacking the N-terminal D1 domain (S1_87–557_, Figure 1) was tested *in vivo*. Upon overexpression of the C-terminally FLAG-tagged native S1 protein or the S1_87–557_ variant in *E. coli* strain JE28, ribosomes were isolated and the ribosome free S100 extract was prepared. The presence of native S1 and its variant on 70 S ribosomes and in the S100 extract was determined by western blot analysis. The result shown in Figure 2C reveals that in contrast to the native S1 (Figure 2C, panel a, lanes 1 and 2), protein S1_87–557_ does not interact with the ribosome, as it can be detected solely in the ribosome free S100 fraction (Figure 2C, panel a, lanes 3 and 4). This result supports the notion that the interaction with the ribosome occurs within the first 86 amino acid residues of protein S1.

### Domain D2 is not involved in ribosome binding of protein S1

**Figure 3:**
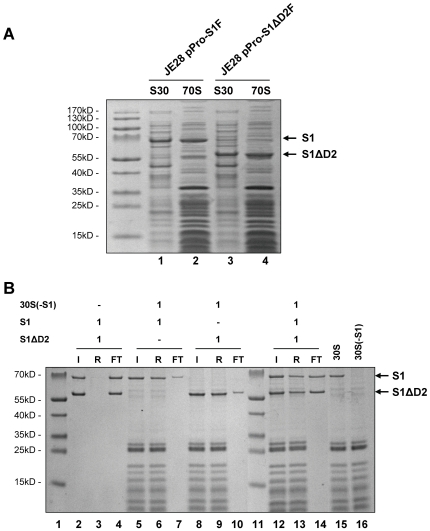
Domain D2 is not involved in ribosome binding of protein S1. (**A**) Ribosome binding of proteins S1 or S1_Δ103–181_ was determined 60 minutes upon induction of their synthesis in strain JE28 harbouring either plasmid pPro-S1F (lanes 1 and 2) or pPro-S1ΔD2F (lanes 3 and 4). S30 extracts (lanes 1 and 3) and purified 70 S ribosomes (lanes 2 and 4) were loaded on SDS-PAGE. The positions of proteins S1 and S1_Δ103–181_ are indicated to the right. (**B**) The binding of S1 (lanes 5–7) or S1_Δ103–181_ (lanes 8–10) for the ribosome was determined by *in vitro* reconstitution experiments employing 30 S(-S1) subunits. The affinity of both proteins was directly compared by a competition experiment incubating 30 S (-S1) ribosomes concomitantly with both proteins S1 and S1_Δ103–181_ in equimolar amounts (lanes 12–14). Upon incubation the ribosomes were separated from unbound proteins as described in Material and Methods, and the proteins present in the different fractions were separated on SDS-PAGE and visualized by Coomassie staining. I, input (lanes 2, 5, 8, and 12); R, ribosome fractions (lanes 3, 6, 9, and 13); FT, flow through fractions (lanes 4, 7, 10, and 14). 30 S, 30 S ribosomes before depletion for protein S1 (lane 15); 30 S(-S1), S1 depleted ribosomes used for the study (lane 16). The positions of proteins S1 and S1_Δ103–181_ are indicated to the right. Lanes 1 and 11, protein size marker.

To scrutinize whether domain D2 might provide secondary contacts with ribosomal proteins at the platform of the 30 subunit, which might possibly enhance the affinity of the protein, assembly of another S1 variant lacking domain D2, here referred to as protein S1_Δ103–181_ (Figure 1), was determined *in vivo* and *in vitro* (Figure 3). Upon induction of S1_Δ103–181_ synthesis in strain JE28 harbouring plasmid pPro-S1ΔD2F, 70 S ribosomes were purified, and binding of protein S1_Δ103–181_ to the ribosome was determined by SDS-PAGE (Figure 3A). The results revealed that the amount of protein S1_Δ103–181_ bound to the 70 S ribosome (lane 4) *in vivo* is comparable to the amount of native S1 (lane 2), and concomitantly, the amount of protein S1 is reduced (lane 4), indicating that S1_Δ103–181_ binds to the ribosome and displaces S1 *in vivo*. To directly compare the ribosome affinity of both S1 and S1_Δ103–181_, we performed *in vitro* reconstitution experiments employing 30 S ribosomes depleted for S1 (30 S(-S1)) and purified proteins S1 and S1_Δ103–181_. Upon incubation of the 30 S(-S1) subunits with the respective S1 proteins, the ribosome fraction was separated from unbound S1 proteins by ultrafiltration as specified in Materials and Methods. The results shown in Figure 3B revealed that protein S1_Δ103–181_ binds in stoichiometric amounts to the ribosome (Figure 3B, lane 9) comparable to binding of native protein S1 (Figure 3B, lane 6). Moreover, in competition assays when proteins S1 and S1_Δ103–181_ were added concomitantly in equimolar amounts to 30 S(-S1) subunits, both proteins bound in a 1∶1 ratio to the ribosomes (Figure 3B, lane 13), indicating that the lack of domain D2 does not reduce the affinity of the protein for the 30 S(-S1) subunit.

### Protein S1_106_ inhibits translation of canonical mRNAs but does not affect lmRNA translation

**Figure 4:**
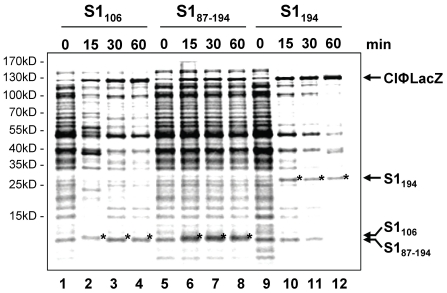
Synthesis of S1 variants S1_106_ and S1_194_ results in selective translation of lmRNAs. Pulse labelling of strain JE28 harbouring plasmids encoding proteins S1_106_ (lanes 1–4), S1_87–194_ (lanes 5–8), and S1_194_ (lanes 9–12) was carried out before (time point 0) and 15, 30, and 60 min upon induction as described in Materials and Methods. Labelled proteins were separated on a 12.5% SDS-PAGE. Positions of proteins S1_106_, S1_87–194_ and S1_194_ (marked by asterisks) and the position of the CI-LacZ fusion protein encoded by a lmRNA are indicated to the right of the autoradiograph.

Taken together, these results reveal that domain D1 interacts with the ribosome and subsequently prevents binding of native protein S1. As S1 is essential for translation initiation on canonical mRNAs [2] we rationalized that overexpression of domain D1 might inhibit translation of canonical mRNAs. Since translation of lmRNA can be accomplished in the absence of protein S1 [9], [10], we thus asked whether overexpression of protein S1_106_ could render the translational apparatus selective for lmRNAs. Therefore, translation was monitored *in vivo* upon overexpression of proteins S1_106_, S1_87–194_ and S1_194_ by pulse labelling. Briefly, *E. coli* strains JE28 harbouring plasmid pKTplaccI (encoding the leaderless *cI-lacZ* fusion gene) [28] and either plasmid pPro-S1D1-2F, pPro-S1D1F or pPro-S1D2F (encoding proteins S1_194_, S1_106_, and S1_87–194_; Table 1), respectively, were grown in M9 minimal medium and pulse labelling was performed before and 15, 30, and 60 minutes after addition of IPTG as specified in Materials and Methods. As shown in Figure 4, the synthesis of protein S1_87–194_, representing domain D2, did not affect translation of bulk mRNA (lanes 5–8). However, upon induction of synthesis of proteins S1_106_ and S1_194_ translation of bulk mRNA ceased, whereas translation of the leaderless *c*I-*lacZ* mRNA continued (lanes 2–4 and lanes 10–11). To ensure translation of proteins S1_106_, S1_194_, and S1_87–194_ (Figure 3, indicated by stars) under these conditions the respective transcripts harbour an unstructured leader of 17 nucleotides in length containing a SD-sequence, translation of which likewise does not require protein S1, as revealed by toeprinting analysis [9].

### Protein S1_106_ interacts with protein S2

**Figure 5:**
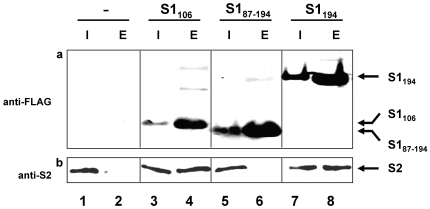
S1 variants S1_106_ and S1_194_ directly interact with protein S2. S30 extracts (Input, I; lanes 1, 3, 5, and 7) prepared from *E. coli* strain Tuner harbouring the empty vector pProEX-HTb (lanes 1 and 2) and its derivatives encoding proteins S1_106_ (lanes 3 and 4), S1_87–194_ (lanes 5 and 6) and S1_194_ (lanes 7 and 8) were loaded onto Ni-NTA agarose to allow binding of the HIS-tagged S1 variants. After washing with 10 column volumes the proteins bound to the matrix were eluted (Elution, E; lanes 2, 4, 6, and 8). The presence of protein S1 variants in input and elution fractions was checked employing anti-FLAG antibodies (panel a). Likewise, both fractions were assayed for the co-purification of protein S2 by SDS-PAGE followed by western blot analysis employing an anti-S2 antibody (panel b).

Collectively, the results shown above indicated that the N-terminal domain is required for S1 binding to the ribosome. Since several lines of evidence reveal that S1 assembly is mediated by direct interaction with protein S2 [10], [23] and moreover, a stoichiometric S1–S2 complex was identified by co-purification with the RNA-polymerase [24] and by immunoprecipitation [25], we next addressed the question, whether only domain D1 of protein S1 is pivotal for this interaction. To this end we performed a pull down assay employing the tagged protein S1 variants. Briefly, *E. coli* strains Tuner harbouring plasmid pProEX-S1D1-2F, pProEX-S1D1F or pProEX-S1D2F (encoding proteins S1_194_, S1_106_, and S1_87–194_ containing an N-terminal HIS-Tag and C-terminal FLAG-tag; Table 1), respectively, were grown in LB medium. Upon overexpression of S1 variants, S30 extracts were prepared and loaded onto a Ni-NTA agarose column to allow binding of the tagged proteins S1_106_, S1_87–194_ and S1_194_. After vigorous washing, the proteins bound to the column were eluted and tested for co-purification of protein S2 by western blot analysis. As shown in Figure 5, concomitantly with the elution of proteins S1_106_ and S1_194_ (panel b, lanes 4 and 8) we obtained a significant amount of endogenous protein S2 (panel a, lanes 4 and 8). In contrast, we did not observe co-purification of protein S2 when protein S1_87–194_ was bound to the Ni-NTA matrix (Figure 5, panel a and b, lane 6), which lacks the N-terminal D1 domain. Taken together, these data support the notion that solely domain D1 is involved in direct interaction with protein S2. These results were supported by far-western blotting (Figure S1A and Information S1) and a yeast two hybrid approach (Figure S1B, a–e), which likewise indicated the interaction between protein S1 and its variants, S1_106_ and S1_194_, with S2 (Figure S1 and Information S1).

### The coiled-coil domain of protein S2 is sufficient to allow recruitment of protein S1 to the ribosome

**Figure 6:**
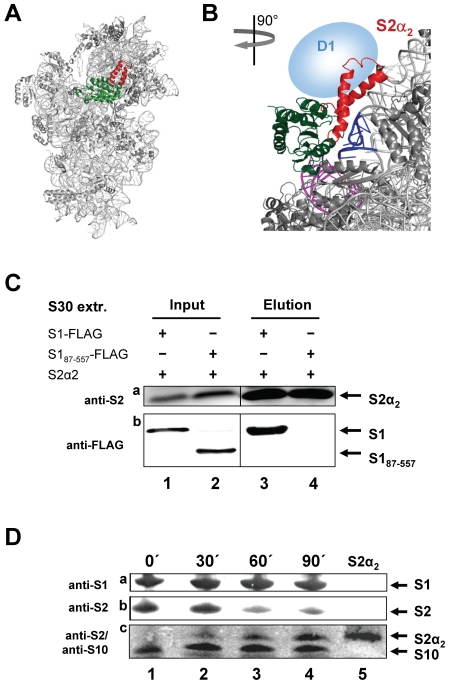
Protein S1 interacts with the coiled-coil domain of S2 *via* its N-terminal domain. (**A**) Position of protein S2 on the 30 S subunit. The structure of the 30 S subunit was modelled employing Polyview 3D [46] and PyMOL molecular system software [47] and PDB file 2AVY [48]. The 16 S rRNA and proteins are shown in light and dark grey, respectively. The globular domain and the coiled-coil domain of S2 are indicated in green and red, respectively. (**B**) Enlargement and clockwise rotation of the structure shown in (A). The coiled-coil domain of protein S2 (S2α_2_; red) interacts with helices h35–h37 (blue) within the head of the 30 S subunit, whereas the globular domain (green) contacts helix h26 (magenta) in the body of the 30 S subunit. The tentative position of domain D1 of protein S1 interacting with the S2α_2_ domain is indicated by a blue sphere. (**C**) S30 extracts containing either FLAG-tagged proteins S1, S1_87–557_, or the HIS-tagged protein S2α_2_ were mixed. An aliquot was subjected to western blot analysis to determine the amount of respective proteins present (lanes 1 and 2). Then the mixture was applied to a Ni-NTA column. Upon washing protein S2α_2_ was eluted and the co-purification of S1 (lane 3) or S1_87–557_ (lane 4) was checked by western blotting using anti-FLAG antibodies (panel b). The amount of protein S2α_2_ was determined using anti-S2 antibodies (panel a). (**D**) 70 S ribosomes were purified from *E. coli* strain Tuner (DE3) before (lane 1; time point 0) and 30, 60 and 90 min upon induction of protein S2α_2_ synthesis (lanes 2–4). The presence of proteins S1, S2, and S2α_2_ was determined by western blotting using anti-S1 (panel a) and anti-S2 antibodies (panels b and c), respectively. The amount of protein S10, which served as an internal control was determined by anti-S10 antibodies (panel c). Lane 5, purified S2α_2_ was loaded to unambiguously identify the protein.

During the analysis of the crystal structure of the 30 S ribosomal subunit the structure of ribosomal protein S2 was determined [29]. The protein is located at the solvent side of the 30 S subunit at the hinge region between the head and the body of the particle [29]. As shown in Figure 6, the protein consists of a large globular domain (indicated in green) and a protruding coiled-coil domain spanning amino acids 110–150 (S2α_2_; indicated in red), which are connected by an unstructured neck region (Figure 6A and B). The globular domain of protein S2 is functionally implicated in accommodation and stabilization of the SD-aSD duplex in the post-initiation complex [30], whereas the side of the coiled-coil protrusion S2α_2_ mediates the interaction with helices 35–37 of the 16 S rRNA [29].

Considering the proposed localization of protein S1 on the 30 S ribosomal subunit by Sengupta *et al.* [22], which indicates that the long arm of protein S1 (LA), representing the N-terminal domain, lies in close proximity to the S2α_2_ domain, we next tested the direct interaction between these domains as specified in Materials and Methods. Briefly, S30 extracts prepared from *E. coli* Tuner cells over-producing the HIS-tagged S2α_2_ domain was mixed with S30 extracts prepared from cells over-producing either the FLAG-tagged protein S1 or the FLAG-tagged protein S1_87–557_, and incubated with Ni-NTA agarose to allow binding of the S2α_2_ domain. After several washing steps to remove unspecifically bound proteins, protein S2α_2_ and its potential binding partners were eluted by addition of imidazole. Western blot analysis of the elution fractions revealed that only full length protein S1 co-purifies with protein S2α_2_ (Figure 6C, panel a, lane 2). In contrast, we did not detect protein S1_87–557_, lacking the N-terminal domain D1 upon elution of S2α_2_ (Figure 6C, panel a, lane 4). Taken together, this analysis strongly supports the notion that the domain D1 of protein S1 is required for direct interaction with the coiled coil domain of protein S2. In addition, the yeast-two hybrid system mentioned above likewise revealed a direct interaction between proteins S1_106_ and S2α_2_ (Figure S1B, f and g and Supporting Information S1).

Since most interactions between S2 and the 16 S rRNA are formed *via* the coiled-coil domain [29], [30], we anticipated that overexpression of the S2α_2_ domain could outcompete native protein S2 from the ribosome. However, taking the interaction between S1 and the S2α_2_ domain into account, it seemed conceivable that binding of S2α_2_ would not interfere with assembly of protein S1 to the 30 S subunit, as it could provide the platform for S1 binding. In order to test for this hypothesis, *E. coli* strain Tuner harbouring plasmid pET-ccS2, encoding the S2α_2_ domain (Table 1) was grown in LB broth. Ribosomes were purified by sucrose density gradient centrifugation as specified in Materials and Methods, before (time point 0) and 30, 60, and 90 minutes upon addition of IPTG to induce synthesis of the coiled-coil domain of protein S2. The presence of native S1 and S2 proteins as well as of the S2α_2_ domain on crude ribosomes was determined employing antibodies directed against proteins S1 and S2. As shown in Figure 6D, upon induction of S2α_2_ synthesis, we were able to verify binding of the S2α_2_ domain to the ribosome (panel c, lanes 2–4). Concomitantly, the amount of native protein S2 bound to the ribosome was severely reduced (panel b, lanes 2–4). As expected the amount of protein S1 remained constant during the course of the experiment (panel a, lanes 1–4). Surprisingly, despite the presence of protein S1 on the ribosome overexpression of the S2α_2_ domain severely affected cell growth (data not shown). Recently, structural analyses indicated that the globular domain of protein S2 lies in close proximity to the SD helix [30], [31]. Thus, our data could be explained by an essential function of the globular domain in the formation and correct orientation of the SD-aSD helix in the post-initiation complex. Further experiments addressing this hypothesis are currently in progress.

## Discussion

### Protein S1 binds to the coiled-coil domain of protein S2 *via* its N-terminal domain D1 on and off the ribosome

In spite of the detailed structural analysis of the *E. coli* ribosome at atomic resolution, the precise site where protein S1 interacts with the ribosome still remains to be elucidated. Almost 30 years ago, it has been suggested that protein S1 associates with the 30 S ribosomal subunit by means of protein-protein interaction [32] mediated by the two N-terminal domains of S1 [12], [33]. Previous biochemical studies and cross-linking experiments indicated that protein S1 is located in spatial proximity to proteins S2, S10, and S18 [20], [21]. These results are consistent with the observation that incorporation of ribosomal protein S2 is pivotal for binding of protein S1 [10], [23]. Moreover, the formation of a stoichiometric complex between proteins S1 and S2 independent of the ribosome was determined by co-purification with the RNA-polymerase [24]. In addition, this complex was reported to regulate expression of the *rpsB-tsf* operon encoding protein S2 and elongation factor EF-Ts [25]. Here, we were able to narrow down the interaction surface between proteins S1 and S2 to the N-terminal domain D1 of S1 and the coiled-coil domain of protein S2 (S2α_2_). We show that (i) deletion of domain D1 abrogates interaction of protein S1 with the ribosome *in vivo* (Figure 2C), and (ii) synthesis of protein S1_106_ is toxic for *E. coli* bacteria (Figure 2A) as it binds to ribosomes and thus prevents assembly of native protein S1 (Figure 2B). These results are in agreement with the fact that domain D1 is absent from S1 proteins of Gram-positive bacteria with a low GC content, where S1 is not a true component of the ribosome and is not essential for protein synthesis [18], [34]. In addition, we provide evidence that deletion of domain D2 did not reduce the affinity of S1 to the ribosome (Figure 3). However, the synthesis of protein S1_Δ103–181_ severely reduces bacterial growth (data not shown) indicating an essential function intrinsic to domain D2, which still remains to be elucidated. Recently, a function in recognition of the tmRNA required for the translational quality-control process of trans-translation was proposed for the second S1 domain [15]. Thus, it might be feasible that the lack of domain D2 could interfere with binding of tmRNA to the ribosome and thereby prevent the rescue of ribosomes stalled on defective mRNAs. However, since the function and essentiality of protein S1 in trans-translation is still a matter of debate and controversy [35], [36], and furthermore, the lack of tmRNA does not severely affect cell growth at 37°C in LB [37], the second S1 domain could potentially provide an intrinsic flexibility to protein S1 that is necessary for its function in translation initiation.

Taken together, our data imply a potential model for the assembly of S1 to the ribosome, wherein the first domain of the protein interacts primarily with the ribosome *via* the S2α_2_ domain. Subsequently, the protein might be accommodated on the 30 S subunit at the platform near proteins S10 and S18, considering their close proximity revealed by cross-linking analysis [21]. Moreover, the fact that interaction with the small subunit occurs only *via* domain D1, could allow a high degree of flexibility to domains D2–D6 of the protein, which might be required to reach out into the solvent to bind structured mRNAs upstream of their rbs in order to recruit them to the ribosome [3], [4], [5], [16].

### A potential role for S1 in translation regulation by ribosome heterogeneity?

In Gram-negative bacteria protein S1 is an essential mediator in translation initiation [2]. It binds to the 5′-UTR of mRNAs, at regions rich in pyrimidines upstream of the rbs. Its role is thought to unwind secondary structures within translation initiation regions in order to facilitate translation initiation complex formation and recognition of the correct start codon with the aid of three initiation factors [6], [7], [38]. Moreover, a possible role for protein S1 in fidelity of translation elongation was proposed [39], and Pedersen and co-workers suggested that S1-deficient ribosomes are inactive in peptide chain elongation in *E. coli* [2]. In contrast, depletion of S1 from crude extracts by anti-S1 serum was shown not to affect translation elongation [40]. In addition, translation of lmRNAs can be accomplished in the absence of protein S1 [10]. Both observations indicate the dispensability of S1 in translation elongation. The current work supports this notion, as translation of lmRNA in contrast to bulk mRNA continues upon induction of S1_106_ synthesis *in vivo*, implying that translation elongation is not affected by replacement of native protein S1 by its truncated variant (Figure 4). Collectively, these data confirm the view that lack of S1 confers selectivity for lmRNAs to the translational machinery. Considering our recent finding that lmRNAs are generated under stress conditions by the endonucleolytic activity of the toxin component of the *mazEF* toxin-anti-toxin module [41], it is tempting to speculate that the selectivity of the translational machinery for lmRNAs could likewise be modulated by presence or absence of protein S1. In support of this notion, recent data indicate that under normal physiological conditions a subpopulation of ribosomes lacking S1 might be present in *E. coli* cells [42]. The authors have shown that overexpression of *rpsA*, encoding protein S1, results in removal of lmRNAs from ribosomes, and depletion of S1 increases the amount of lmRNAs in the ribosome fraction. Thus, it is tempting to speculate that distinct physiological conditions might increase the amount of S1-depleted ribosomes and thereby stimulate specific translation of lmRNAs. To this end, one could envisage depletion of S1 from the ribosome by interfering with its assembly, potentially by blocking the S1–S2 interaction described here. Experiments addressing this hypothesis are currently performed.

## Materials and Methods

### Bacterial strains and plasmids

*E. coli* strains, plasmids and oligonucleotides used in this study are listed in Tables 1 and 2. Unless otherwise indicated, bacterial cultures were grown at 37°C in LB medium [43] supplemented with ampicillin (100 µg/ml) or kanamycin (20 µg/ml) where appropriate. Growth was monitored by measuring the optical density at 600 nm (OD_600_).

**Table 2:** Synthetic oligonucleotides used in this study.

	Sequence*	Restriction sites	Binding region
**B5fw**	TATA*GGCGCCGAATTC* ATGACTGAATCTTTTGCTC	*Nar*I, *EcoR*I,	*rpsA* from codon 1
**D5fw**	TATA*GGCGCCGAATTC* ATGAAAGCTAAACGTCAC	*Nar*I, *EcoR*I,	*rpsA* from codon 87
**G5rev**	TATA*CTCGAG*TTA**TTTTTCATCGTCATCCTTATAGTC**AGCATCTTCGTAAGC	*Xho*I	*rpsA* until codon 106
**H5rev**	TATA*CTCGAG*TTA**TTTTTCATCGTCATCCTTATAGTCCATGCCTTCCTGCAGGGTC**CATGCCTTCCTGCAGG	*Xho*I	*rpsA* until codon 194
**I5rev**	TATA*CTCGAG*TTA**TTTTTCATCGTCATCCTTATAGTC**GCCTTTAGCTGCTTTG	*Xho*I	*rpsA* until codon 557
**V14fw**	[P]GCCGTTATCGAATCCGAAAAC		*rpsA* from codon 182
**W14rev**	[P]GTAAGCTTTTTCCAGCGTGATCC		*rpsA* until codon 102
**J5**	TATA*GAATTCCTCGAG* GGTCTGTTTCCTGTG	*EcoR*I, *Xho*I	pProEX-Htb specific primer used for site-directed mutagenesis
**H4fw**	TATA*CATATG* AACCATCGCTGGCTGG	*Nde*I	*rpsB* from codon 93
**I4rev**	TATA*CTCGAG* TTAGTCCGGCAGACCGC	*Xho*I	*rpsB* until codon 159

* Restriction sites are highlighted in italics; sequences encoding the FLAG-tag are shown in bold, and sequences complementary to the template are underlined.

### Construction of plasmids

Coding sequences of protein S1 and its variants were amplified by PCR employing primers indicated in Table 2. The PCR products were digested with *NarI* and *XhoI* and ligated into the corresponding sites of pProEX-HTb (Invitrogen). To remove the HIS-tag sequence, the pProEX-HTb derivatives were amplified using the plasmid-specific primer J5 (Table 2) and the respective forward primer (B5 or D5; Table 2). The PCR products were digested with *EcoR*I and *Dpn*I and ligated by T4 DNA ligase (Fermentas). This procedure resulted in pProEX-HTb derivatives lacking the sequence encoding for the N-terminal HIS-tag followed by TEV-cleavage site (Table 1; pPro plasmids). Plasmids pProEX-S1F and pPro-S1F have been used for creating plasmids pProEX-S1ΔD2F and pPro-S1ΔD2F using site-directed mutagenesis kit (NEB) and 5′-monophosphorylated primers V14 and W14. The coding sequence of protein S2α_2_ (S2_93–159_) was amplified by PCR employing primers H4 and I4 (Table 2). The PCR product was digested with *Nde*I and *Xho*I and ligated into the corresponding sites of pET22b (Novagen). All plasmid constructs were verified by sequencing (AGOWA).

### Ribosome purification employing the Ni-NTA agarose

*E. coli* JE28 strains harbouring plasmids pPro-S1D1-2F, pPro-S1D1F, pPro-S1D2F and pPro-S1ΔD2 (encoding proteins S1_194_, S1_106_, S1_87–194_ and S1_Δ103–181_; Table 1) were grown in LB broth in the presence of 100 µg/ml ampicillin and 20 µg/ml kanamycin. At OD_600_ 0.20–0.25 synthesis of protein S1 variants was induced by addition of 50 µM IPTG. 60 minutes upon induction cells were harvested by centrifugation and lysed by the freeze-thaw method in lysis buffer (20 mM Tris·HCl, pH 7.4, 10 mM MgCl_2_, 30 mM NH_4_Cl, 100 mM KCl, 10 mM Imidazole, 1 u/ml RNase-free DNase I (Roche)). After centrifugation at 30 000 g, S30 extracts were applied to a Ni-NTA agarose column, washed by 10 column volumes of washing buffer (20 mM Tris·HCl, pH 7.4, 10 mM MgCl_2_, 30 mM NH_4_Cl, 150 mM KCl, 20 mM Imidazole) followed by elution of 70 S ribosomes with elution buffer (20 mM Tris·HCl, pH 7.4, 10 mM MgCl_2_, 30 mM NH_4_Cl, 150 mM KCl, 150 mM Imidazole). The protein composition of ribosomes was determined by separation of equimolar amounts of ribosomes by SDS-PAGE followed by western blot analysis using antibodies against ribosomal proteins.

### Ribosome purification employing a sucrose cushion

*E. coli* strain Tuner (DE3) harbouring plasmid pET-ccS2 (encoding protein S2_93–159_ encompassing the coiled-coil domain of S2; Table 1) was grown in LB broth in the presence of 100 µg/ml ampicillin. At OD_600_ of 0.25–0.3 the synthesis of protein S2α_2_ was induced by addition of 100 µM IPTG. Before (time point 0) and 30, 60, and 90 minutes upon addition of IPTG, 200 ml aliquots were withdrawn, harvested by centrifugation and lysed by the freeze-thaw method in lysis buffer (20 mM Tris·HCl, pH 7.4, 10 mM MgCl_2_, 30 mM NH_4_Cl, 100 mM KCl, 1 u/ml RNase-free DNase I (Roche)). After centrifugation at 30.000 g, S30 extracts were layered on top of the sucrose cushion (20 mM Tris·HCl, pH 7.4, 10 mM MgCl_2_, 300 mM NH_4_Cl, 100 mM KCl, 1.1 M Sucrose) followed by centrifugation at 100 000 g. Pelleted ribosomes were resuspended in resuspension buffer (20 mM Tris·HCl, pH 7.4, 10 mM MgCl_2_, 30 mM NH_4_Cl, 10 µM β-mercaptoethanol). The protein composition of ribosomes was determined by separation of equimolar amounts of ribosomes by SDS-PAGE followed by Western blot analysis using antibodies against ribosomal proteins.

### Preparation of 30 S ribosomal subunits depleted of protein S1 (30 S(-S1))

30 S subunits were prepared as described before [44] and were depleted for protein S1 by affinity chromatography using poly(U)-Sepharose 4B (GE Healthcare) as described elsewhere [45].

### Purification of proteins S1 and S1_Δ103–181_

*E. coli* strain Tuner has been transformed with plasmids pProEX-S1F and pProEX-S1ΔD2. The cultures have been grown until OD_600_ 0.3–0.4. Protein over production has been induced by addition of 100 µM IPTG. After 4 hrs the cells were harvested by centrifugation and resuspended in the Lysis Buffer (20 mM HEPES•KOH pH7.6, 6 mM MgCl_2_, 30 mM NH_4_Cl, 200 mM KCl, 5 mM imidazole). Cells were disrupted by sonication. His-tagged proteins were purified using TALON-resin (Clonetech) according to the manufacturer's protocol followed by size-exclusion chromatography on Sephadex S200 (GE Healthcare) in 20 mM HEPES•KOH pH7.6, 200 mM KCl. Finally, the proteins were dialyzed against 1× TICO buffer.

### In vitro reconstitution of 30 S subunits

20 pmoles of 30 S(-S1) subunits were incubated at 37°C for 30 min in the presence of a 1∶1 molar ratio of purified proteins S1 and S1_Δ103–181_ as indicated in Figure 3B. Then the ribosome fraction was separated from the free proteins employing Amicon ultrafiltration membrane with MWCO 100 kDa (Millipore). The retained fractions were washed twice with 1× TICO buffer. The flow through was adjusted to the volume of the initial reaction mixture (50 µl) using Amicon ultrafiltration membrane with MWCO 3 kDa (Millipore). The protein composition of the Input (I), ribosome (R), and flow through (FT) fractions were analyzed using SDS-PAGE.

### *De novo* synthesis of the CI-LacZ protein upon overexpression of protein S1 variants

*E. coli* JE28 strains harbouring plasmid pKTplaccI [26] along with plasmids pProEX-HTb, pPro-S1D1-2F, pPro-S1D1F or pPro-S1D2F were grown in M9 minimal medium in the presence of 100 µg/ml ampicillin and 20 µg/ml kanamycin. At OD_600_ of 0.2–0.25, expression of S1 variants was induced by addition of 50 µM IPTG. Before and at time points 15, 30, and 60 min after IPTG addition, aliquots were withdrawn and pulse labelling was carried out for 5 min at 37°C essentially as described before [44]. The reactions were stopped by addition of an equal volume of cold 10% TCA, followed by incubation in ice for 15 min and subsequent centrifugation for 15 min at 15 000 rpm at 4°C. The pellets were washed with 90% acetone, dried under vacuum, and resuspended in SDS-protein sample buffer. Prior to loading onto a 12% SDS-polyacrylamide gel, the samples were denatured at 95°C for 5 min. For the different OD_600_ values, the same amounts of total cellular proteins were subjected to electrophoresis. The gels were dried and exposed to a Molecular Dynamics PhosphoImager for visualization and quantification.

### Determination of the interaction between protein S1 variants and protein S2

*E. coli* Tuner cells containing plasmids pProEX-HTb, pProEX-S1D1-2F, pProEX-S1D1F or pProEX-S1D2F were grown in LB broth in the presence of ampicillin 100 µg/ml to OD_600_ of 0.20–0.25. The synthesis of protein variants was induced by addition of 50 µM IPTG. 60 minutes upon induction cells were harvested by centrifugation and lysed by the freezing-thawing method in lysis buffer (50 mM Na_2_HPO_4_, pH 8.0, 300 mM NaCl, 10 mM Imidazole, 0.1% Tween-20, 0.5 mg/ml DNase I (Roche), 20 µg/ml RNase A). After centrifugation, extracts were applied to Ni-NTA agarose columns, washed by 10 column volumes of washing buffer (50 mM Na_2_HPO_4_, pH 8.0, 500 mM NaCl, 20 mM Imidazole) and proteins were eluted with elution buffer (50 mM Na_2_HPO_4_, pH 8.0, 300 mM NaCl, 250 mM Imidazole). Protein concentrations were determined employing a Bradford assay. The proteins present in the eluted fractions were separated by SDS-PAGE followed by Western blot analysis using antibodies specific for ribosomal proteins.

### Determination of the interaction between S1 and S1_87–557_ proteins and protein S2α_2_

*E. coli* Tuner cells containing plasmids pPro-S1F and pPro-S1ΔD1F and Tuner (DE3) cells harbouring plasmid pET-ccS2 were grown in LB broth in the presence of 100 µg/ml ampicillin. At an OD_600_ of 0.25–0.30 the synthesis of protein variants was induced by the addition of 100 µM IPTG, and 60 minutes thereafter the cells were harvested by centrifugation and lysed by the freezing-thawing method in lysis buffer (50 mM Na_2_HPO_4_, pH 8.0, 300 mM NaCl, 10 mM Imidazole, 0.1% Tween-20, 0.5 mg/ml DNase I (Roche), 20 µg/ml RNase A. After centrifugation, the extract containing protein S2α_2_ was split into two parts. Each part was mixed with the extract containing either protein S1-FLAG or S1_87–557_-FLAG. Obtained mixtures were applied to Ni-NTA agarose columns, washed by 10 column volumes of washing buffer (50 mM Na_2_HPO_4_, pH 8.0, 500 mM NaCl, 20 mM Imidazole) and proteins were eluted with elution buffer (50 mM Na_2_HPO_4_, pH 8.0, 300 mM NaCl, 250 mM Imidazole). Protein concentrations were determined employing a Bradford assay. The proteins present in the eluted fractions were separated by SDS-PAGE followed by Western blot analysis using antibodies specific for ribosomal proteins.

## Supporting Information

Figure S1 **Far-western blot analysis (A) and yeast two hybrid approach (B) indicating the interaction between protein S1 or its variants and protein S2 or its coiled-coil domain S2α_2_.** (**A**) 2.5 µg of total extract of cells over expressing the SH2-S2 fusion protein were separated on a 12% SDS PAGE and transferred to nitrocellulose membranes. After renaturation, the membranes were individually incubated with different concentrations of purified S1_106_ (lanes 1–3) and S1 proteins (lanes 5–7): (lanes 1 and 7: 300 µg/ml, lanes 2 and 6: 30 µg/ml, lanes 3 and 5: 3 µg/ml), respectively. Lane 4: no bait protein was added. The blots were probed with anti-S1 antibody. S1_106_ and S1 bound to the SH2-S2 fusion protein and S2 are indicated by an open and a closed arrow, respectively. The positions of the bands corresponding to SH2-S2 and native S2 protein were verified by probing the same membranes with anti-S2 antibody (lane C). Two signals that were also present in the absence of the bait proteins are likely detected due to non-specific binding of anti-S1-antibody to other polypeptides or to proteolysis forms of endogenous protein S1 (marked with closed circles). (**B**) The β-galactosidase activity given in Miller units (MU) was used as reporter for the protein-protein interactions. a and b: controls lacking one interaction partner. c, d and e: MU representing interaction between proteins S1_106_, S1_194_ and native S1 with protein S2, respectively. f and g: Interaction between native S1 or S1_106_ and the coiled-coil domain of protein S2, respectively.
(TIF)

Information S1 **Supplement for Materials and Methods section (PDF).**
(PDF)
