# Supplementary material for: Direct Interaction of the N-Terminal Domain of Ribosomal Protein S1 with Protein S2 in Escherichia coli
Source: PLoS One. 2012 Mar 7;7(3):e32702. doi: 10.1371/journal.pone.0032702 (PMC3296737; doi:10.1371/journal.pone.0032702)
Supplement: Information S1 — Supplement for Materials and Methods section (PDF). (PDF) [file pone.0032702.s002.pdf]

Supporting Information S1: Supplement for Materials and Methods

of the article: *Byrgazov et al. 2011.* ***Direct Interaction of the N-Terminal Domain of Ribosomal Protein S1 with Protein S2 in Escherichia coli***, published at PLoS ONE

Studying the interaction between ribosomal proteins S1 and S2 and variants thereof employing Far-western blotting and yeast two hybrid analyses

Far-western blot analysis

To determine the interaction between proteins S1 or $S1_{106}$ and S2 using Far-western blotting, 2.5 µg total cell extracts from *E. coli* cells harbouring plasmid pET42-SH2-S2 encoding a SH2-S2 fusion protein (generated to study protein-protein interaction by NMR Probing [1]) were prepared upon induction of SH2-S2 protein synthesis and separated on a 12% SDS PAGE. The proteins were transferred to a nitrocellulose membrane, and the membrane was washed with 1x PBS to remove SDS. The proteins bound to the membrane were refolded by incubation in 6M and then 3M Guanidine-HCl in AC buffer (10% glycerol, 100mM NaCl, 20 mM Tris, 0.5 mM EDTA, 1mM DTT and 0.1% Tween-20) supplemented with 2% milk powder for 30 min at room temperature. Then, the blot was washed with 1M (and then 0.1M) Guanidine-HCl in AC buffer supplemented with 2% milk powder for 30 min at 4ºC. The membrane was quickly rinsed in AC buffer and blocked in AC buffer containing 2% skim milk at 4ºC overnight. After incubation of the membrane with 300, 30, 3 µg/ml or without bait proteins (protein S1 or $S1_{106}$) at 4ºC for 2-3 hours, the membrane was washed twice wash with AC buffer for 10 min at room temperature and further incubated with anti-S1 antibody in AC buffer with 2% milk. After two times washing with AC buffer for 10 min at room temperature, the membrane was incubated for 30 min with the secondary antibody conjugated with alkaline phosphatase and further visualized using 5-bromo-4-chloro-3-indolylphosphate (BCIP) and nitroblue tetrayolium (NBT) as chromogenic substrates.

Construction of plasmids used in the yeast-two hybrid analysis

To scrutinize the protein-protein interactions between S1 and S2 *in vivo*, a yeast two hybrid system was employed. The plasmids, pGBT9 (harbouring the Gal4 DNA-binding domain, DBD; Table S1) and pGAD424 (harbouring the Gal4 activation domain, AD; Table S1) were used for the studies. Briefly, genes encoding ribosomal proteins S1 and its variants $S1_{106}$ and

$S1_{194}$ were amplified by PCR using primer pairs FPY2HS1/RPY2HS1, FPY2HS1/RPY2HS1106 and FPY2HS1/RPY2HS1194 (Table S2), respectively. The PCR products were digested with enzymes *EcoR*I/*BamH*I and ligated into the corresponding site of plasmid pGBT9, thereby creating plasmids pGBTS1, $pGBTS1_{106}$, and $pGBTS1_{194}$, respectively (Table S1). Likewise, the gene encoding protein S2 was amplified with primer pair FPY2HS2/RPY2HS2, and primer pair FPY2HS2helix/RPY2HS2helix was used to amplify the coiled-coil domain $S2\alpha_2$. The PCR products were digested with *EcoR*I/*BamH*I and cloned into plasmid pGAD424 to give rise to plasmids pGADS2 and pGADS2α2, respectively (Table S1).

Analysis of protein interactions using a yeast two hybrid system

The yeast two hybrid system experiments were performed according to manufacturer's manual (Clontech, US). Briefly, yeast strain HF7c harbouring the corresponding bait and prey plasmid combinations (Table S1) was grown in YPD medium or in synthetic dropout medium lacking Leu, Trp or both (Clontech, US). β-galactosidase was used as reporter for the protein-protein interactions, and β-galactosidase assays from yeast cells were performed as described before [2]. The assays were performed in triplicate.

References

[1] Ludwiczek, M.L., Baminger, B., and Konrat, R. (2004). NMR probing of protein-protein interactions using reporter ligands and affinity tags. J Am Chem Soc *126*, 1636-1637.

[2] Guarente, L. (1983). Yeast promoters and lacZ fusions designed to study expression of cloned genes in yeast. Methods Enzymol 101, 181-191.

[3] Kaberdina A.-C., PhD Thesis (2010) Studies on the formation of ribosome heterogeneity and its function on translation initiation in *Escherichia coli.*

Table S1. Strains and plasmids used in the Far western blot analysis and the yeast two hybrid analysis.

	**Genotype/Relevant features**	**Source/Reference**
**Yeast srain:**		
HF7c	*MAT**a** ura3-52 his3-Δ200 trp1-Δ901 leu2-3, 112 lys2-801 ade2-101 gal4-542 gal80-538 LYS2::GAL1-HIS3 URA3::(GAL4 17mers)*$_3$*-CYC1-lacZ*	Clontech, US
**Plasmids:**		
pET42-SH2-S2	pET42b containing the SH2-S2 fusion gene	[3]
pGBT9	*GAL4*-DBD; *TRP1* Amp^r	Clontech, US
pGAD424	*GAL4*-AD; *LEU2* Amp^r	Clontech, US
pGBTS1	pGBT9-derivative encoding the GAL4-DBD-S1 fusion protein	This work
pGBTS1$_{106}$	pGBT9-derivative encoding the GAL4-DBD-S1$_{106}$ fusion protein	This work
pGBTS1$_{194}$	pGBT9-derivative encoding the GAL4-DBD-S1$_{194}$ fusion protein	This work
pGADS2	pGAD424-derivative encoding the GAL4-AD-S2 fusion protein	This work
pGADS2α2	pGAD424-derivative encoding the GAL4-AD-S2α_2 fusion protein	This work

Table S2. Synthetic oligonucleotides used in this study

Name	Sequence*
FPY2HS1	AATT**GGATCCGAATTC**ATGACTGAATCTTTTGCTCA
FPY2HS2	AATT**GGATCCGAATTC**ATGGCAACTGTTTCCA
FPY2HS2helix	AATT**GGATCCGAATTC**ATGCTGACTAACTGGAAAACC
RPY2HS1	AATT**GGATCCCTGCAG**TTACTCGCCTTTAGCTG
RPY2HS1106	AATT**GGATCCCTGCAG**TTATTCAGCATCTTCGTAAG
RPY2HS1194	AATT**GGATCCCTGCAG**TTATTCCATGCCTTCC
RPY2HS2	AATT**GGATCCCTGCAG**TTACTCAGCTTCTACGAAGC
RPY2HS2helix	AATT**GGATCCCTGCAG**TTAGCCCAGGCTGTTTTCCAG

*Restriction sites are highlighted in bold type and the sequence complementary to the template is underlined
